# Role of Social Support in Women facing Domestic Violence during Lockdown of Covid-19 while Cohabiting with the Abusers: Analysis of Cases Registered with the Family Counseling Centre, Alwar, India

**DOI:** 10.1177/0192513X20984496

**Published:** 2021-11

**Authors:** Meerambika Mahapatro, Moksh M. Prasad, Sudhir Pratap Singh

**Affiliations:** 1 National Institute of Health and Family Welfare, New Delhi, India; 2 JSS Medical College, Mysuru, Karnataka, India; 3 Management Development Institute, Gurgaon and Sapna, Alwar

**Keywords:** domestic violence, COVID-19, India, social support, coping, mental health, family counseling center

## Abstract

The present study aims to analyze the role of social support in the lives of women survivors of domestic violence who filed a complaint with the Mahila Salah and Suraksha Kendra (MSSK) Alwar, India, while residing with the abusive husband and his family during the lockdown period of COVID-19. The study explores the role of MSSK with extended vulnerability of women during the lockdown period at large. This study adopts an exploratory qualitative method. A total of 36 married women who had filed a complaint with MSSK before and during the lockdown were included. Interviews with the women were held through telephonic conversations on vulnerability, coping mechanism and extent and forms of social support. Thematic content analysis was done in a stepwise manner. Results show that degeneration of social support model is time -bound and the accuracy of applying this model wane under extended condition of vulnerability caused due to COVID-19. MSSK can expand support by creating and integrating virtual community networks to detect and deter violence during the lockdown. The study suggests that the government can ensure and empower bystanders with skills of modern communication. The existing physical institutional delivery mechanism need to evolve strategies that are resilient to emerging threats from the vulnerable ecosystem.

## Introduction

The current pandemic of COVID-19 has led the world into a frenzy. Practicing safe hygiene, social distancing, and travel restrictions have become common sights throughout the globe. Effects of COVID-19 include a spike in domestic violence (DV) and is presumed to be seen continually across the globe as stress continues to mount (Peterman et al., 2020; Weitzman & Behrman, 2016). Many victims of DV are still trapped with the perpetrators, with no means to report it. International studies have shown that women are more likely to face violence when confined with little or no support of law enforcement agencies due to the lockdown (Campbell et al., 2017; UN News, 2020). The Government of India announced a nationwide lockdown from March 25 to May 17, 2020, to contain the spread of the disease. This unprecedented crisis has led to rapidly increasing stress, sudden shifts in daily routines, unemployment, alcohol abuse, and a rapid onset of scarcity in the availability of essential commodities alongside limited access to social support systems. All of these have been identified as risk factors of DV globally (Devries et al, 2013; Zahran et al., 2009). Learning from past pandemics, the risk of serious psychological consequences increases with the increase in the duration of quarantine (Brooks et al., 2020). Additionally, the accumulation of stressful events poses a risk of significant physical and/or emotional harm (Campbell et al., 2017; Catalá-Miñana et al, 2017). These adverse effects may extend for long periods due to continued abuse, ongoing psychological effects of abuse, or fear of the abuser (Stewart & Vigod, 2017). It is a frequently reported behavior in abusers to try to isolate and control their victims so as they may not report violence (Mahapatro, 2018). As the mobility of the victim is restricted and the perpetrator can easily control access to social media and other means of possible reportage, it is next to impossible for the victim to reach out for help. Places of worship and communal places of congregation that were used for finding emotional reprieve are inaccessible during current times (Gelder et al., 2020). As a consequence of controlling behavior by the abusers, mental distress increases and may range from heightened stress, frustration, and anger to severe depression and post-traumatic stress disorder (Fulu et al, 2013). Suicide has been reported following the imposition of quarantine in previous outbreaks (Barbisch et al., 2015). The situation is forcing people to remain confined to their homes with limited access to essential services and minimized social support options. Current situations further enable the abuser to easily hush the victim.

Social support has been found to both mediate (Beeble et al., 2009) and/or moderate (Kaslow et al., 1998) the relationship between intimate partner violence (IPV) and mental health. Social support can be interpreted as social capital (Putman, 1995). It is an important intervention during stressful events in the family and the individual’s life (Cooke et al., 1998). Under undue stress, social support is key in relieving the victim’s distress. Research has identified the beneficial effects of social support on women’s overall mental health (Ferrari et al., 2016; Harandi, 2017). Having stronger family support increases the strength to deal with psychological distress as well as with the abuse. A woman staying with an abusive husband and his family is twice more likely to develop psychological distress compared to a woman having the support of her parents (Mahapatro & Singh, 2019). Adequate social support decreases the risk of violence in a relationship and its negative impacts if present (Cohen et al., 2000; Katerndahl et al., 2013). The perception that one has access to informational, emotional, psychological, financial, and/or instrumental aid has been associated with positive health outcomes (Lindsay & Yates, 2004). Emotional support by friends and family prevents deterioration of mental health (Bosch & Bergen, 2006; Coker et al., 2002) by them acting as a buffer or moderator to provide a positive impact regardless of the severity of abuse (Cohen et al., 2000; Meadows et al., 2005;). The interactions and dynamics of social support are complex, but they always show a positive correlation with the quality of life and negative correlation with the extent of depression in the victims (Beeble et al., 2009).

Longitudinal studies carried out among the abused women in an Australian shelter have shown that social support in the lives of women causes a substantial reduction in posttraumatic stress disorder, depression, and anxiety (Martin & Mohr, 2001), whereas ongoing abuse and the absence of social support contributes to psychological distress. Another longitudinal study by Sullivan and colleagues found that higher social support was related to decreased abuse and higher quality of life at multiple points in time of the study (Bybee & Sullivan, 2002). Abused women who receive emotional, tangible, and institutional support are less vulnerable to psychologically damaging effects of violence and their physical safety is also maintained (Panaghi et al., 2013). Social support influences coping strategies and provides greater perceived options that contribute to increasing the feasibility of a battered woman’s ability to deal with abusers. Earlier studies have reported that social support works in both ways, directly promoting recovery from stressful experiences and crises experienced in the family as well as the protective role of a buffer against the effects of life stressors (House 1981). A study conducted by Mahapatro and Singh (2019) revealed that women who were supported by their parental family moved on to engage in active coping. It was also reported that they needed more social support, particularly from their parental family. They said that an institution could only provide temporary relief from fear and anxiety, but support from the parental family was permanent. In India, the concept of a woman seeking formal support and institutional help is not welcomed by the community. Therefore, the government-designed and implemented institutional programs have often not been utilized optimally. It is only the natal family that extends support to the abused women.

## The Context and the Problem

In the Indian context, after getting married, when women move to the husband’s family, it is seen as a detachment and social isolation from her kinship and natal family. In a situation such as this, where her support system has considerably narrowed (Turner & Marino, 1994), she is expected to forgo her established social capital and strive to adjust in every circumstance with her limited social support. In Indian cultures, women are seen as more of a collective unit of society, and social support is conceptualized as an interdependent culture and a transaction of sorts in which one person seeks help from another (Taylor et al., 2004).

At the time of crisis, when the women are in distress due to DV, they are left with meager social support to cope with violence they are subjected to (Mahapatro & Singh, 2019). Therefore, the investigation of social support is a prerequisite for social and mental resilience during family violence. In India, DV is recognized by the law, predominantly experienced by women, and characterized by “physical, sexual, or psychological harm by the husband or his family members in a domestic sphere” (*The Gazette of India*, 2005). The DV Act of 2005 recognizes the legal rights of women and has established a decentralized model of Family Counseling Centers (FCCs) in every district of India. The FCC is implemented in collaboration with the police, judiciary, NGOs, and other departments throughout the country (Singh & Mahapatro, 2018). Alwar district headquarters has a Mahila Salah and Suraksha Kendra (MSSK), an FCC that addresses different elements of awareness and women empowerment. The centre, with its women-centric and culture-sensitive approach, has improved the utilization of legal remedies and providing psychological assistance in an integrated approach for better coping. In the absence of social support, these interventional programs are the only remaining instruments to create a positive impact by translating the abuse experienced by individual women into a cumulative human-rights discourse. In these processes of intervention, the victims are actively engaged and made empowered in order to utilize different methods of coping (Mahapatro & Singh, 2019). The institutional support to women survivors of DV is an important policy instrument for improving the survivors’ well-being, especially in an unsupportive social context. The abused women register their complaint with these centers while residing with the abusive husband and their family, and they need not to move out of the home.

During COVID-19, coping strategies utilized by battered women registered with MSSK and residing with the abuser allowed several inferences to be made. Previous studies have reported that every year, about 200 DV cases are registered with the MSSK of Alwar district (Singh & Mahapatro, 2018). The MSSK was closed during the lockdown and started operating again on April 20, 2020, under orders of the government. Due to limited mobility during the lockdown, the MSSK could operate solely through mobile phones. However, the country-wide use of this intervention provided a unique opportunity to further investigate the situation during the lockdown period. The present study aims to analyze the situation regarding the role of social support to women survivors who filed a complaint with the MSSK Alwar, India, while residing with the abusive husband and his family members during the COVID-19. Further, the study explores the role of MSSK during the period of lockdown.

## Methods

Study Design: The study adopted an exploratory qualitative method. Ethical approval for the study was obtained from SAPNA, the NGO running the FCC at Alwar.

Study area/setting: The MSSK, one of the FCCs serving women who have suffered domestic violence located in Alwar, Rajasthan, was selected for this study.

Study population and sample size: The study participants were composed of married women facing abuse who had filed a complaint with the MSSK, and who were staying with the abusive husband and his family and wished to continue the marriage. Those women who had filed for divorce were excluded because they had not been staying with the husband or his family. With the help of the register and records maintained by the counselors at MSSK, the women participants were selected. A total of 36 women who had registered with MSSK from January to April 2020 were considered; while 6 new cases (March 23–April 15, 2020) during the first lock down, and 33 earlier registered cases (January to March 22, 2020) were followed up. Only three women were excluded as they were not able to be reached, one of those interviewed had terminated the marriage or registered under divorce. MSSK was open to physical visits and registering complaints after April 15, 2020. The interviews were conducted in the month of April 2020 telephonically.

## Data Collection

The interview schedule was open-ended in nature and structured to examine four key determinants: incidence of DV, role of MSSK, social support available to them, and the coping mechanisms provided to them, in order to deal with their traumatic experiences. The qualitative nature of these interviews helped to glean critical insights into the interviewees’ perception. Moreover, each woman respondent’s attitude and sensitivity to dealing with everyday situations at home was also assessed.

Interviews with women were held only after obtaining their verbal consent. The women were approached by the female counselors of MSSK over the phone. They had to be telephoned several times before they could be reached. A few limitations that were encountered in virtual communication included fear of a lack of privacy and confidentiality. In many cases, women were reluctant to answer questions about violence that they deemed unimportant in comparison to their immediate concerns regarding food, money, health, and the prevailing situation. Some women refused to answer questions over the phone and wanted to talk through a physical confrontation in MSSK only. However, measures were taken to minimize the risk of this non-response bias by allowing respondents to choose a suitable date and time. Thereafter, the counselor tried several times to contact the women when they could respond without the fear of their conversation being interrupted or eavesdropped. This was essential to guarantee their safety, apart from preserving the ethics and protocols of research so that respondents were comfortable enough to respond freely. Additionally, as per the government guidelines, follow-up measures were taken by the counselors and they were expected to call each survivor and understand their situation, extend support, and ensure their safety. During the COVID crises, women were not in a mood to talk to the counselors of MSSK. However, the telephonic conversation could not be continued for more than a certain period, with the time of conversation varying between 10 and 20 minutes. Access to information over messaging platforms was limited due to the vulnerability of privacy.

## Data Analysis

Interview data was analyzed using content analysis. In all the cases, individuals were assessed to see whether the social support changes in terms of its form and nature during the lockdown period compared to the usual phenomena. The criteria were determined as previously outlined in the theoretical model of social support (House, 1981). The description of incidental details was recorded in a narrative form. This allowed the perception of women’s experiences of DV in a situational context and helped in examining how the shifting of subjectivity assisted in changing their social support and coping strategies.

## Definitions Used

1. Definition of DV: The study used the definitions of DV as defined in the Domestic Violence Act 2005, India (*The Gazette of India*, 2005), and analyzed under physical, sexual, psychological, or economic violence.
2. Coping: Coping is a process through life where women are capable of combating and using different skills and resources. The term coping was used to mean the processes by which survivors engaged to seek support, redress, and transform some form or forms of damage they had suffered.
3. Social Support: Four types of social support have been studied as per the definition given by House (1981): (a) emotional support (providing empathy, listening, love, trust, and care); (b) instrumental support (providing aid in terms of cash or kind); (c) informational support (providing advice, suggestions, and information related to better coping); and (d) appraisal support (providing affirmation, feedback, social comparison, and self-evaluation).

## Results

Social support and coping with violence during the lockdown

Social support has been studied with the following four factors as given by House (1981). In the current context, some of the situations seen are as follows:

1. **Emotional support:** During the first few months of marriage, the family acts as a source of emotional support. The mother of the woman listens to her empathetically. Yet it is not a permanent solution. One of the women raised a question, “How many times will they listen to the same story? It doesn’t help much after a few months if the violence continues in the same manner.” During the lockdown, an alteration in the form of violence was also seen as physical abuse moderated. Although psychological violence persisted, it did not affect the women as deeply as before because the tension associated with the lockdown overshadowed their personal conflicts. The major factor influencing abuse was the consumption of liquor. As due to the lockdown liquor was unavailable, the severity of violence decreased greatly. Some respondents reported that the husbands were fearing COVID-19 and turned into god-fearing men. Other survivors reconcile their condition as “my individual suffering is not of such a huge amount that I talk about what’s happening in my home, when the whole world is suffering due to corona.” Some respondents reported that they were not getting time with their husbands to discuss issues or to introspect because they would return in the evening in a state of inebriation and exhibit violent behavior. In the absence of alcohol, as they could spend more time together, communication improved, and they got time to introspect and discuss issues with their husbands. Even if their husbands gave them a little time to voice themselves and talk about issues, they felt elated.
2. **Instrumental support:** Generally, while getting married, the natal family offers monetary support to their daughter in the form of a part of their property or money. But in many cases, as the parents of the woman themselves belong to middle and low-income families, they can’t provide much to support their daughters. Some respondents reported that their parents support a divorce so that they can have a better life with the alimony as compared to their unbearable present condition. However, they find it difficult to make any final decision that does not have a detrimental effect on their children’s future.
3. **Informational support:** Parents try to advise and guide their daughter, but it doesn’t help much because of a marked variation in the context and situation vary. Mostly women said that since it was an unprecedented crisis with limited options and information asymmetry, a sense of fear regarding the family’s well-being had developed in the abusers, which made them spiritually inclined and helped them cope better.
4. **Appraisal support:** The natal family keeps trying to soothe things to absorb the pain, tolerate the torture, and try to uphold the woman’s morale. It does help get through the situation, but it does not change the situation. It is often the parents of the survivor who have helped them in reporting the case to the authorities. Additionally, it is not a common practice for bystanders or neighbors to report cases to the authority. As stated by a survivor, “when I am trapped in the house with my violent husband, it severely limits contact with my natal family and the outside world. With the scenario being the ‘worst-case’ possible where there is a threat to our lives, I am left with no option but to forgive and ignore as much as possible.” Another survivor reported, “I keep things to myself as there are other things to think about such as how to run the household when it has become so difficult to get things during the lockdown.”

These observations point out that the model is applicable over a relatively shorter duration of time. As the length of abuse extends for years, the rigidity in applying the model starts decreasing. In the present situation, as the woman is not economically independent and instrumental support is largely absent, social support stands for a relatively short duration after which due to long term abuse it falters. Regarding emotional and appraisal support, it is reported that the mother of the abused woman is the primary support giver. In the current time of lockdown, the woman is not able to contact her primary support giver, her mother, as the perpetrator is always around and has access to any and every means of communication, and the freedom to express herself is low. As the woman has no other option but to reside with the perpetrator, lodging a complaint with the institution remains the only form of assurance to the victim. Else, if the situation is not sufferable by the victim, she can choose to reside in the institution, an implication of which is publicly declaring her absence of any form of social support. In such a situation, the matter becomes publicly known and neighbors, friends, and family get word of it, but the victim is at least given a chance to thrive at the institution. A common concern for the women is coping during the lockdown with limited social capital available to them. It was inquired whether the victims shared their problems with their close friends and their natal families. They said that even if they did inform their friends and family, there is hardly anything they could do except worry. With the change in form, nature, and severity of DV during COVID-19, the role of social support and coping strategy hasn’t changed to a great extent.

The women who approach MSSK learn about the other victims who have been suffering. It provides a means for social comparison and self-evaluation that acts as a form of appraisal support from the institution. It also gives them a sense of security seeing other women in a similar situation approaching the institution during their time of need. The institution then becomes an important place to find support and develop coping mechanisms as more than the institution itself the victims place their trust on the people representing the institution. Women reported that physical access to MSSK provided a higher degree of emotional satisfaction, yet the steps taken by the MSSK during the lockdown restrictions, had still provided them some form of social support to deal with their problems. For the women, even these subtle changes in the behavior of their husbands gave them a sense of positive well-being. However, during the current time of crises, the women are unable to approach the MSSK and are not able to see the standard for social comparison and are not able to evaluate themselves.

## Role of MSSK during Lockdown

The lockdown has limited the MSSK to communicate with the women through mobile phones, which lacks compassionate and continuous counseling. With the constant presence of family members, and without face-to-face confidence-building interaction, mobile phones can only provide emergency services for women, especially for women reporting violence for the first time. Although the MSSK counselors appeal to the registered victims to check their well-being, they were unable to bring out refined personalized narratives due to paucity of time and the possibility of breach of privacy. As the husband is constantly at home, access to the phone is not easy. The women do not prefer messaging platforms because it leaves behind evidence. This has further decreased reportage. The counsellor reported that even if they wished to help the survivor, they could not do much as their details including phone numbers and files were in the office of the centre, the calls are received at the centre, and until the lockdown eased the staff did not have access to the records outside the centre.

## Discussion

The pandemic has led to an imbalance in various factors and a new social equilibrium. Changes in certain perceptions and gross increase in risk factors is causing a surge in the number of cases pertaining to DV. The pandemic created social acceptance to psychological abuse, with an increased risk of developing anxiety and depression. Social support is a dynamic perception that changes with the extent and gravity of the problem along with the support that a woman’s natal family and friends can deliver at the time of crisis (Bosch & Bergen, 2006). In the initial days of conflict and abuse by the husband and his family members, support is offered by the natal family, particularly her mother who listens to her pain and provides emotional support. As the duration of the lockdown extends along with the risk of abuse, the natal family is still willing to provide support, but the victim’s need for social support exceeds what is available to them, thus decreasing their ability to cope using the available social support. House’s (1981) model of social support doesn’t work to a great extent if the time period is lengthened substantially. If the situation is likely to continue, vulnerability due to the COVID-19 crisis increases and the model starts to lose its efficacy. Since the types of support available to the women readily are emotional, informational, and appraisal, without its instrumental form, the perceived support of the women makes them feel hopeless, suggesting higher dependency on institutional support as it is tangible. The mediation period was interminable and traumatic; the institutional support to women survivors of DV was an important policy instrument for improving survivors’ well-being. Parson (2010) reported similar results in his study of battered women. With the change in nature of problem during COVID-19, degeneration of social support is a time-bound phenomenon in the absence of instrumental support, and its waning or buffering action isn’t affected much.

In the women’s point of view, majority of the coping strategies were based on problem focused approaches where the central cause of stress was the abusive partner or relation. By resolving various peripheral possible causes of conflict, the women used to deal with the stressor. A shift is being seen as the husband spends more time to reflect and retrospect with the woman, which leads to resolution of minute differences and decreases the risk of violence. When the husband spends even a little bit of time listening to the women’s problems and matters of the home that they want to discuss with them, the women feel a sense of elation. This is supplemented by the gross reduction in alcohol consumption. Alcohol is a recurrent theme that largely contributes to violence due to the alterations in behavior that it causes (Mahapatro, 2018). Extended time spent at home helped partners to cope better under the lockdown. Spiritual inclination of the husband due to fear of the pandemic and other fears associated with the financial crisis eased day to day differences. Few of the women reported that “the extent of violence before the lockdown set in was so severe that the amount of abuse during lockdown doesn’t feel as much because we can understand his situation.” Some others reported that “the little time given by my husband during the lockdown, time he had not given me otherwise, felt very gratifying.” The husbands tried to transform their outlook to reassure themselves in these trying times. This shift is helping decrease the peripheral burden of coping.

Women think that disengaging from the abusive husband or leaving the abusive husband are not the only solutions. Upon leaving abusive partners, women have to encounter a number of challenges including navigating through many transitions, which bring with them potential for increased tension or conflict. Such changes are not always welcome by society. In reality, the women don’t have a buffet of avenues to cope; most of the times they choose to forgive the abuser to avoid confrontation and drown themselves in household chores to forget the instances of abuse. Another more favorable aspect of emotional coping for women is the formation of social networks. It’s a positive step instead of justifying abuse and habituating it. Women who struggle in their current social networks in addition to coping with ongoing conflicts with the husband and his family, might not benefit from the usual interventions of social support. The reason may also be attributed to the fact that women have less social support and are neither well adept nor have the privacy to use information and communication technology to reach out for help.

Some limitations of the study are attributed to the telephonic conversations. As the time duration on the call is short, responses were short and interviews were concise. Some were using their husbands’ phones and, due to the presence of other family members, a reporting bias may have crept in, making it difficult to draw conclusions on the state of mental distress and severity of violence suffered by the women during the lockdown.

The interventions from MSSK proved to be transformative for the survivors in the society as they spoke of this process as both the transformation of the self and giving rise to the embodiment of social transformation through social exchange. Similarly, other studies reported that online or telephonic support helps decrease anxiety, stress, and depression when the identity is not disclosed. It acts as a buffer to protect individuals from different aspects, particularly against certain life stressors. The paper highlights how traditional physical institutional arrangements have ceased due to the lockdown that has been imposed. An analysis of the need for a dynamic, virtual structure to adapt to the existing ecosystem is necessary. With the lockdown in place, both the victims and MSSK are struggling to find a channel for communication on both ends. Trying to reach out to them over the phone doesn’t provide much of a solution as the perpetrators are constantly present; accessing the phone or even finding enough privacy to report the situation accurately, is difficult. During times like pandemics, people start developing a sense of unease towards the system. The physical presence of the counselor provides continuous counseling with compassion that builds confidence. The mobile phone is only able to provide emergency services for women (Mahaptro & Singh, 2019). It is crucial to create a new instrument or device to access services from the MSSK.

On the whole, the study suggests that the psychological impacts of lockdown and being trapped with the abusers are wide-ranging, long-lasting, and unabating. The results suggest that the government should take the initiative to ensure their safety and well-being. This may be achieved by means of awareness campaigns and of dissemination of coping and stress management techniques to combat boredom, activation of social networks, and emotional help and counseling through telecommunication. MSSK should augment its infrastructure by integrating virtual community networks through emerging technology to monitor violence and follow up cases during the lockdown. Another concern is that of bystanders and neighbors. Instilling a sense of moral and social responsibility will improve reportage (Campbell, 2020). Frontline workers such as postal workers, garbage collectors, food delivery persons, and home appliances repair persons, and people who visit the family and discern DV, should come forward and report violence in the current times. The MSSK’s interventions can further rebuild social networks of women to address the conflicts and reduce vulnerability with timely access of rights while seeking justice. The findings of the paper suggest that existing institutional arrangements need to evolve strategies that are resilient to emerging threats in the existing ecosystem.
